# Glial Cysteine Cathepsins: From Homeostasis to Neurodegeneration

**DOI:** 10.1007/s10571-026-01776-w

**Published:** 2026-07-09

**Authors:** Anja Suhadolc, Selena Horvat, Janko Kos, Anja Pišlar

**Affiliations:** 1 Department of Pharmaceutical Biology, Faculty of Pharmacy, University of Ljubljana, Aškerčeva cesta 7, 1000 Ljubljana, Slovenia; 2 Department of Biotechnology, Jožef Stefan Institute, Jamova cesta 39, 1000 Ljubljana, Slovenia

**Keywords:** Glial cells, Neuroinflammation, Neurodegeneration, Cysteine cathepsins, Therapeutic targets

## Abstract

Glial cells, namely microglia, astrocytes, and oligodendrocytes, play crucial roles in maintaining homeostasis in the central nervous system and orchestrating responses to injury, infection, and disease. Among the molecular regulators of glial function, cysteine cathepsins have emerged as key modulators of both physiological and pathological processes. These lysosomal peptidases are traditionally known for their housekeeping roles in protein degradation; however, accumulating evidence highlights their broader involvement in antigen presentation, microglial and astrocyte reactivity, inflammatory signalling, apoptosis, and myelination. Under normal conditions, cysteine cathepsins support essential functions in the central nervous system, including immune surveillance and tissue remodelling. Conversely, their dysregulation, characterized by overexpression, increased enzymatic activity, or mislocalization, can promote neuroinflammation and neurodegeneration, contributing to the pathogenesis of disorders such as Alzheimer’s disease and multiple sclerosis. This review provides a comprehensive synthesis specifically focused on the diverse roles of cysteine cathepsins across major glial cell types, systematically summarizing current knowledge in microglia, astrocytes, and oligodendrocytes. We emphasize their cell type-specific, context-dependent, protective, and deleterious functions. Furthermore, we discuss mechanistic links between cysteine cathepsin activity and neurodegenerative processes and evaluate the therapeutic potential and current limitations of selectively targeting glial cysteine cathepsins. A deeper understanding of the context-dependent dual roles of these enzymes in brain physiology and pathology is critical for designing targeted interventions that could mitigate neuroinflammation and neurodegeneration.

**Graphical Abstract:**

**Figure:**
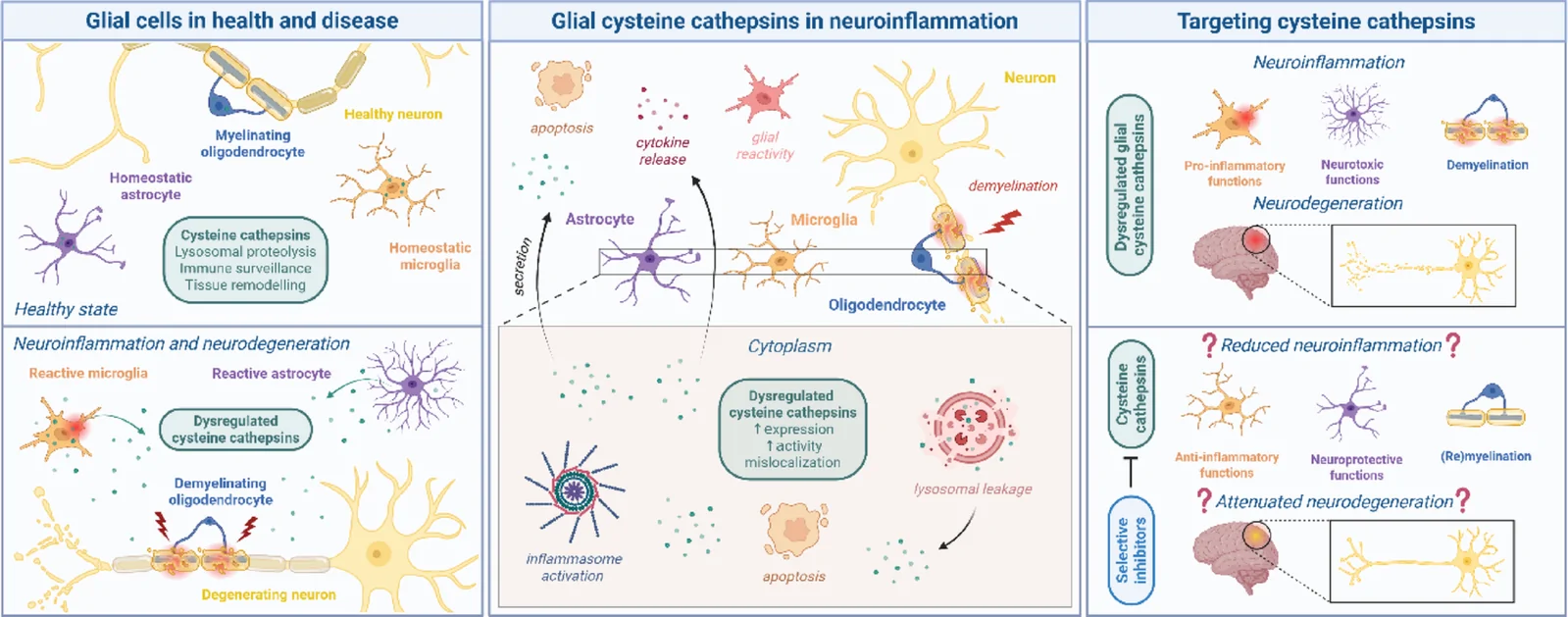

## Introduction

The central nervous system (CNS) consists of two cell types: neuronal and glial cells. The latter are distributed throughout the brain and constitute approximately half of the cells of the CNS (reviewed in von Bartheld 2016). Although these neuron-supporting cells were once considered to be static bystanders, it is now well established that their diverse and dynamic functions orchestrate nearly every aspect of CNS formation, development, and function (reviewed in Allen and Lyons 2018). Beyond their physiological functions, glial cells are also critically involved in CNS pathology, including major neurodegenerative diseases (reviewed in Roth et al. 2005; González et al. 2014; Tang and Le 2016; Du et al. 2017; Kwon and Koh 2020; Fan and Huo 2021; Boccazzi et al. 2022; Bhusal et al. 2023; Patani et al. 2023; Zveik et al. 2024). Among the many regulators of glial cell function, cysteine cathepsins have emerged as important contributors (reviewed in Reiser et al. 2010; Pišlar and Kos 2014; Lowry and Klegeris 2018; Nakanishi 2020a; Pišlar et al. 2021; Stoka et al. 2023). While their role in neuroinflammation and neurodegeneration has been addressed in previous reviews, their functions in glial cells have not yet been examined in a systematic and comprehensive manner. Here, we provide an integrative overview of cysteine cathepsins in glial cell biology, emphasizing their context-dependent roles in both physiology and pathology, with particular attention to their involvement in neuroinflammation. Additionally, we critically discuss their potential as therapeutic targets in neuroinflammation and neurodegeneration.

## Physiological and Pathological Roles of Glial Cells in the CNS

**Fig. 1:**
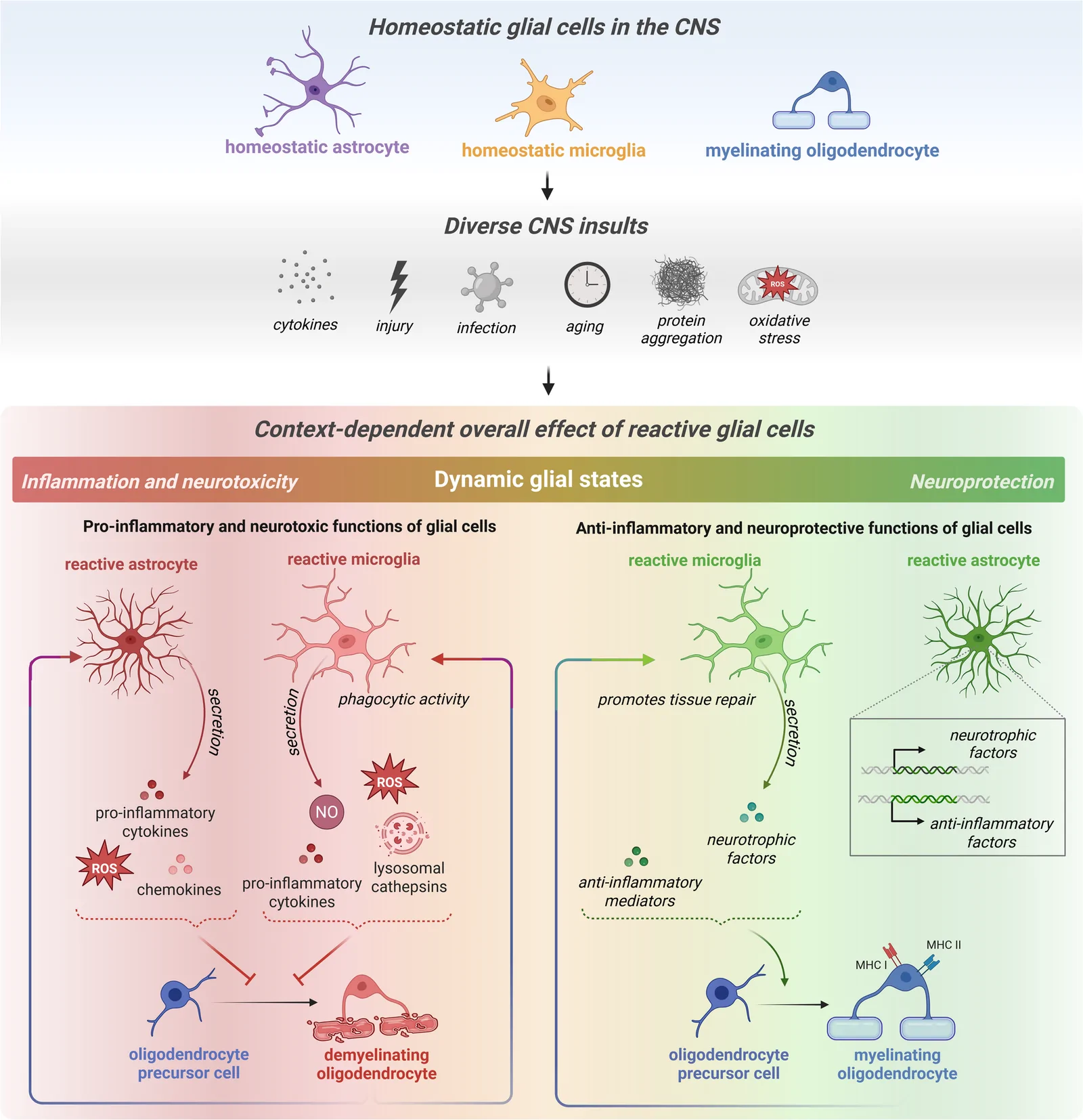
Context-dependent glial reactivity in neuroinflammation. In response to their surrounding cellular environment, microglia and astrocytes adopt heterogeneous and dynamic reactive states with diverse inflammatory, immune, supportive, or reparative functions. These functions influence oligodendrocyte survival, demyelination, and remyelination, thereby shaping neural circuit function. Oligodendrocyte precursor cells and mature oligodendrocytes actively contribute to the neuroimmune environment by expressing immune-related receptors and secreting cytokines. Notably, the overall effect, either neurotoxicity or neuroprotection, depends on the balance and nature of lost and gained homeostatic, protective or detrimental functions, and the relative abundance of different cell populations. MHC: major histocompatibility complex, NO: nitric oxide, ROS: reactive oxygen species. Created with BioRender

Glial cells can be divided into three main groups: microglia, astrocytes, and oligodendrocytes (Fig. 1) (reviewed in Jäkel and Dimou 2017; Allen and Lyons 2018). (1) Microglia are the resident innate immune cells of the brain, essential for immune surveillance within the CNS. In addition to their immunological role, they are involved in synaptic pruning and the clearance of apoptotic neurons (reviewed in Lowry and Klegeris 2018; Allen and Lyons 2018; Peng et al. 2023). (2) Astrocytes perform a wide array of functions, including phagocytosis, synaptic pruning, regulation of homeostasis and maintenance of the blood-brain barrier, as well as providing metabolic and trophic support to neurons (reviewed in Allen and Lyons 2018; Peng et al. 2023). Additionally, although microglia have traditionally been considered the principal immune mediators in the CNS, accumulating evidence also underscores the significant role of astrocytes in modulating immune responses (reviewed in Rawji et al. 2020). (3) Oligodendrocytes form the myelin sheath, enabling rapid signal transmission along axons. They also offer metabolic and trophic support to neurons (reviewed in Allen and Lyons 2018; Peng et al. 2023).

The diverse roles of microglia are largely attributed to their plasticity and ability to adopt heterogeneous and dynamic functional states. Various exogenous and endogenous stimuli, including cytokines, injury, infection, aging, protein aggregation, and oxidative stress, trigger microglial reactivity, marked by altered cell morphology and gene expression (reviewed in Lowry and Klegeris 2018). Traditionally, these responses were often described using binary terms such as “resting” versus “activated” or “classical (M1)” versus “alternative (M2)” phenotypes. The classical (M1) phenotype has been associated with strong phagocytic activity and the secretion of pro-inflammatory cytokines, nitric oxide, reactive oxygen species (ROS), and lysosomal peptidases, all of which contribute to neurotoxicity and exacerbate inflammatory processes (reviewed in Tang and Le 2016; Du et al. 2017). By contrast, the alternative (M2) phenotype has been linked to tissue repair and the secretion of anti-inflammatory mediators and neurotrophic factors that foster a neuroprotective microenvironment and help restore homeostasis (reviewed in González et al. 2014; Tang and Le 2016). However, these classifications are now considered oversimplified and misleading, as single-cell technologies have demonstrated that microglia exist in diverse, dynamic, and context-dependent states rather than fixed polarized phenotypes, with no state being exclusively protective or detrimental (Fig. 1) (Ransohoff 2016; Paolicelli et al. 2022). In particular, recent transcriptomic studies have identified disease-associated microglia (DAM) as a distinct functional state linked to neurodegeneration, further emphasising the complexity and context specificity of microglial responses (Ou et al. 2026).

Similarly, astrocytes undergo a transformation known as reactive astrogliosis following CNS insults (reviewed in Singh 2022). Traditionally, and analogous to the M1/M2 nomenclature for microglia, two major astrocyte phenotypes have often been described: neurotoxic A1 and neuroprotective A2 (reviewed in Zuchero and Barres 2015; Liddelow et al. 2017; Fan and Huo 2021; Patani et al. 2023). A1 astrocytes have been associated with the loss of homeostatic supportive functions and acquisition of harmful properties, including the secretion of pro-inflammatory cytokines, chemokines, and ROS (Zamanian et al. 2012; Liddelow et al. 2017; Fan and Huo 2021; Patani et al. 2023). Conversely, the A2 phenotype has been associated with the upregulation of genes encoding neurotrophic and anti-inflammatory factors, which may promote neuronal survival and tissue remodelling (reviewed in Zamanian et al. 2012; Liddelow et al. 2017; Fan and Huo 2021). Nonetheless, current consensus recommendations emphasize that, like microglia, reactive astrocytes should not be reduced to binary A1/A2 categories, as astrocyte reactivity comprises heterogeneous, context-dependent, and disease-specific states that should be defined by molecular, spatial, temporal, and functional criteria, and where different states may co-exist (Fig. 1) (Escartin et al. 2021). Accordingly, in this review, the M1/M2 and A1/A2 terminology is used only when referring to older studies that originally applied it, whereas microglial and astrocyte responses are otherwise described as homeostatic, reactive, inflammatory, phagocytic, disease-associated, repair-associated, or context-defined states. Importantly, the overall function of reactive microglia and astrocytes, either neuroprotection or neurotoxicity, depends on the balance between the loss of homeostatic functions and gain of protective or detrimental functions, which is highly context-specific and influenced by the nature and duration of CNS insults as well as the surrounding cellular environment (Fig. 1) (Li et al. 2007; Escartin et al. 2021; Guttenplan et al. 2021; Singh 2022; Paolicelli et al. 2022; Feng et al. 2023; Liang et al. 2023; Zhang et al. 2023a; Gomes et al. 2024; Zou et al. 2024).

While microglia and astrocytes are the principal regulators of immune responses in the CNS, oligodendrocytes are among the main targets of immune-mediated damage. Neuroinflammation disrupts oligodendrocyte function, leading to myelin sheath degradation, impaired remyelination, and subsequent axonal injury and neurodegeneration (Fig. 1). This damage, in turn, exacerbates neuroinflammation, creating a self-perpetuating cycle (Roth et al. 2005; Stone et al. 2017; Nasrabady et al. 2018; Heß et al. 2020; Starost et al. 2020; Franklin and Simons 2022; Klotz et al. 2023; Depp et al. 2023; Cammarota and Boscia 2023; Zveik et al. 2024). Importantly, oligodendrocyte precursor cells and mature oligodendrocytes also actively contribute to the inflammatory response (Boccazzi et al. 2022; Zveik et al. 2024). In response to CNS damage, they express mediators and receptors for immune-related molecules (reviewed in Boccazzi et al. 2022), including major histocompatibility complex (MHC) class I and II molecules (Suzumura et al. 1986; Harrington et al. 2023; Catenacci et al. 2025). Moreover, oligodendrocytes secrete inflammatory cytokines (Ma et al. 2025) and influence microglial state and function (Madsen et al. 2020; Boccazzi et al. 2021; Desu et al. 2021), highlighting their dynamic role in shaping the neuroimmune environment.

Beyond their individual functions, glial cells are engaged in dynamic communication, establishing autocrine feedback, modulating each other’s behaviours, and affecting brain homeostasis (reviewed in Jha et al. 2019; Liddelow et al. 2020; Fan and Huo 2021; Singh 2022; Bhusal et al. 2023; Patani et al. 2023). Alterations in glial function and dysregulation of the glial network have been implicated in several major neurodegenerative diseases, including Alzheimer’s disease (AD), Parkinson’s disease, Huntington’s disease, multiple sclerosis (MS), and amyotrophic lateral sclerosis (reviewed in Zuchero and Barres 2015; Peng et al. 2023). As previously discussed, microglia and astrocytes play pivotal roles in neuroinflammation (González et al. 2014; Kwon and Koh 2020). Neuroinflammation primarily serves as a defence mechanism, protecting the CNS from diverse pathogens and injuries through secreting neurotrophic factors, promoting remyelination, and facilitating the clearance of toxic protein aggregates and cellular debris (Wyss-Coray and Mucke 2002; Simard et al. 2006; Glezer et al. 2007; Goldstein et al. 2016; Pöyhönen et al. 2019). However, chronic inflammation is marked by sustained reactivity of glial cells, increased oxidative stress, mitochondrial dysfunction, and initiation of the apoptotic cascade. Collectively, these processes play a central role in the pathogenesis of various neurodegenerative diseases, contributing to neuronal dysfunction, degeneration, and progressive cell loss (González et al. 2014; Kempuraj et al. 2016; Russo and McGavern 2016; Dorothée 2018; Kwon and Koh 2020; Pišlar et al. 2021). The complex and multifaceted functions of glial cells in both physiological and pathological contexts are regulated by numerous factors. Among these, lysosomal peptidases, particularly cysteine cathepsins, are gaining attention as significant modulators of glial activity and neuroinflammatory responses, highlighting their potential role in the development and progression of neurodegenerative conditions associated with neuroinflammation (reviewed in Reiser et al. 2010; Pišlar and Kos 2014; Lowry and Klegeris 2018; Nakanishi 2020a; Pišlar et al. 2021; Stoka et al. 2023).

## Cysteine Cathepsins in the Glial Lysosomal Proteolytic Network

Cysteine cathepsins are a group of lysosomal peptidases, i.e. hydrolytic enzymes that catalyse the breakdown of proteins (reviewed in Turk et al. 2012; Brix 2013; Pišlar et al. 2021). They belong to the cysteine peptidase clan CA and are widely distributed among living organisms (reviewed in Turk et al. 2012; Rawlings et al. 2018). There are 11 human cysteine cathepsins (B, C, F, H, K, L, O, S, V, X, and W), which share a conserved active site formed by cysteine, histidine, and asparagine residues (Turk et al. 2012; Rawlings et al. 2014, 2018). Their key properties and disease-associated phenotypes are summarized in Table 1. Most cysteine cathepsins (F, K, L, O, S, and V) are predominantly endopeptidases, whereas cathepsins C and X are exclusively exopeptidases, exhibiting aminopeptidase and carboxypeptidase activity, respectively. Furthermore, cathepsins B and H possess both endo- and exopeptidase activity and act as both aminopeptidases and carboxypeptidases (reviewed in Stoka et al. 2005). Some cysteine cathepsins (B, H, L, C, and O) are ubiquitously expressed in human tissues and cells, including the brain, whereas others (F, K, S, V, X, and W) exhibit cell-/tissue-specific expression (reviewed in Kos et al. 2009; Pišlar and Kos 2014; Pišlar et al. 2021). Cysteine cathepsins are synthesized as inactive preproenzymes. Following removal of the signal peptide in the endoplasmic reticulum, procathepsins are transported through the secretory pathway and are typically activated in acidic endolysosomal compartments by proteolytic removal of the propeptide (reviewed in Pišlar et al. 2021). This zymogen-based organization prevents uncontrolled proteolysis during biosynthesis and trafficking, ensuring that full enzymatic activity is mainly acquired within lysosomes. Cathepsin function and stability are generally optimal at a slightly acidic pH, such as that found within lysosomes, and are typically reduced at neutral pH, such as that in the cytosol and extracellular environment. However, notable exceptions include cathepsin S, which maintains stability at neutral to slightly alkaline pH (Turk et al. 2012), and cathepsin B, which retains endopeptidase activity under similar conditions (Musil et al. 1991; Turk et al. 1995; Yoon et al. 2022). In addition to pH, cathepsin activity depends on a reducing environment that maintains the catalytic cysteine residue in an active state. Under physiological conditions, potentially harmful proteolysis is further restricted by lysosomal compartmentalization, limited substrate access, and endogenous inhibitors, particularly cystatins and stefins (reviewed in Turk et al. 1995; Turk et al. 2012; Pišlar and Kos 2014). Under pathological conditions, however, this balance may be disrupted by increased cathepsin expression, altered trafficking, lysosomal membrane permeabilization, secretion, or insufficient inhibitor activity.

Cysteine cathepsins were long considered intracellular enzymes, responsible for non-specific bulk proteolysis in endolysosomal compartments (Turk et al. 2000, 2001). It is now well established that their localization extends beyond endosomes and lysosomes, with evidence supporting their presence in the nucleus, cytoplasm, and extracellular space, where their activity is influenced by local pH, redox conditions, inhibitor availability, and extracellular matrix composition (reviewed in Turk et al. 2012). This broader distribution suggests their involvement in a wide range of physiological and pathophysiological cellular processes (reviewed in Turk et al. 2012). For example, they play vital roles in regulating immune responses (e.g. antigen presentation and cytokine and chemokine processing), neuropeptide and hormone processing, and extracellular matrix (ECM) remodelling (which contributes to, e.g., normal bone turnover) (reviewed in Brix et al. 2008; Reiser et al. 2010; Stoka et al. 2016; Vidak et al. 2019; Pišlar et al. 2021).

All cysteine cathepsins, except cathepsin W, are involved in CNS biology and pathology (reviewed in Kos et al. 2009; Pišlar and Kos 2014; Pišlar et al. 2021). In this review, we focus on the 11 human cysteine cathepsins, while cathepsins D and E are considered only briefly where relevant, as they are aspartyl rather than cysteine peptidases. This distinction is important because, despite their shared lysosomal localization and relevance to CNS pathology, cathepsins D and E differ from cysteine cathepsins in the catalytic mechanism and therefore fall outside the main scope of this review. In the cysteine cathepsin family, proteolytic activity can be altered by pathological abnormalities, and increasing evidence indicates that disruptions in the normal balance of enzymatic activities, namely increased proteolytic activity, is a hallmark of aging and neurodegeneration (reviewed in Nakanishi 2003; Turk 2006). Accordingly, increased cathepsin expression and activity in neuronal and glial cells during neurodegeneration are frequently reported (reviewed in Pišlar and Kos 2014). Several papers have reviewed the role of cysteine cathepsins in CNS biology as well as neuroinflammation, neurodegeneration, and other CNS pathologies (Hook 2006; Haque et al. 2008; Pišlar and Kos 2014; Pišlar et al. 2021). Here, we build on this literature by focusing specifically on cysteine cathepsins in glial cells and by comparing their roles in microglia, astrocytes, and oligodendrocytes. In the following sections, the functions of cysteine cathepsins in individual glial cell types are discussed in more detail. Given that the available evidence is unevenly distributed across these glial populations, we distinguish throughout the review between well-supported mechanisms in microglia, more fragmentary but emerging evidence in astrocytes, and the still limited knowledge on oligodendrocyte-intrinsic cysteine cathepsins. This comparative framework helps avoid overgeneralization and highlights key gaps and priorities for future research.

**Table 1:** Comparative overview of human cysteine cathepsins relevant to CNS and glial biology

Cathepsin	Proteolytic activity	pH/stability profile	Tissue distribution	Representative substrates/functions	Endogenous inhibitors/regulation	Selected disease/knockout phenotype and glial relevance	References
Cathepsin B	Endopeptidase and carboxypeptidase	Acidic optimum; can retain activity at near-neutral pH	Broadly expressed, including CNS cells	Protein turnover, Aβ-related processing/clearance, inflammasome-linked pathways	Cystatins/stefins; compartmentalization; pH	Strong evidence in microglial activation, Aβ metabolism, NLRP3 inflammasome activation, and neuroinflammation; disease models indicate both protective roles in aggregate clearance and detrimental roles in inflammatory injury	(Musil et al. 1991; Halle et al. 2008; Turk et al. 2012; Wu et al. 2017; Yoon et al. 2022; Jiang et al. 2025)
Cathepsin C	Aminopeptidase/dipeptidyl peptidase	Acidic lysosomal activity	Immune-enriched; low in healthy CNS but increased in activated microglia	Activation of immune-cell proteases; inflammatory mediator regulation	Cystatins, especially cystatin F; compartmentalization	Linked to microglial activation, chemokine production, EAE, and demyelination models; cathepsin C knockdown preserves myelin integrity in selected demyelination models	(Stoka et al. 2005; Liang et al. 2016; Shimizu et al. 2017; Zhao et al. 2021)
Cathepsin F	Endopeptidase	Acidic lysosomal activity	More restricted expression; detected in CNS-related datasets	Endolysosomal proteolysis; antigen processing-related functions	Cystatins/stefins; compartmentalization	Limited glial-specific evidence; implicated by omics studies in demyelinating disease contexts, including MOG-associated disease	(Kos et al. 2009; Turk et al. 2012; Pišlar and Kos 2014; Pišlar et al. 2021; Wang et al. 2024)
Cathepsin H	Aminopeptidase and endopeptidase	Acidic lysosomal activity	Broadly expressed	Protein turnover; inflammatory and neurotoxic pathways in selected models	Cystatins/stefins; compartmentalization	Linked to microglia-mediated neuronal injury, EAE onset, and impaired remyelination in demyelination model	(Stoka et al. 2005, 2023; Ma et al. 2007; Okada et al. 2018)
Cathepsin K	Endopeptidase/collagenase-like activity	Acidic activity; strong matrix-degrading capacity	Osteoclast-enriched; also detected in selected CNS/glial contexts	Extracellular matrix degradation; lysosomal proteolysis	Cystatins/stefins; compartmentalization	Limited but relevant evidence in astrocyte and microglial phenotypes	(Brix et al. 2008; Reiser et al. 2010; Dauth et al. 2011; Stoka et al. 2016; Pišlar et al. 2021)
Cathepsin L	Endopeptidase	Acidic lysosomal activity; limited neutral stability	Broadly expressed, including CNS cells	Protein turnover, antigen processing, extracellular matrix degradation, apoptosis-related pathways	Cystatins/stefins; compartmentalization	Evidence in microglial activation, antigen presentation, ischemic injury, and demyelinating disease models	(Gresser et al. 2001; Stoka et al. 2005; Ma et al. 2007; Gu et al. 2015; Zhang et al. 2025a)
Cathepsin O	Endopeptidase	Acidic lysosomal activity	Broad expression reported, but poorly characterized	General lysosomal proteolysis	Cystatins/stefins; compartmentalization	Little direct evidence in glial biology	(Kos et al. 2009; Turk et al. 2012; Pišlar and Kos 2014; Rawlings et al. 2018; Pišlar et al. 2021)
Cathepsin S	Endopeptidase	Acidic optimum but unusually stable at neutral pH	Immune cells, antigen-presenting cells, microglia	Invariant chain processing, antigen presentation, extracellular matrix degradation	Cystatins/stefins; compartmentalization; pH	Strong relevance for microglial antigen presentation, migration, and inflammatory responses; cathepsin S deficiency impairs microglial response in selected injury models	(Kos et al. 2009; Turk et al. 2012; Pišlar and Kos 2014; Rawlings et al. 2018; Pišlar et al. 2021)
Cathepsin V	Endopeptidase	Acidic lysosomal activity	Restricted tissue distribution	Lysosomal and extracellular proteolysis in selected tissues	Cystatins/stefins; compartmentalization	Limited CNS evidence; implicated in demyelination/remyelination-related models	(Kos et al. 2009; Pišlar and Kos 2014; Shimizu et al. 2017; Pišlar et al. 2021)
Cathepsin X/Z	Carboxypeptidase	Acidic lysosomal activity; also detected outside lysosomes in disease	Immune cells, microglia, selected glial contexts	C-terminal processing of proteins, including γ-enolase; cell adhesion/signalling	Cystatins/stefins; compartmentalization	Strong relevance in microglial neuroinflammation and oligodendrocyte differentiation; altered activity has been linked to neurodegenerative and inflammatory CNS models	(Obermajer et al. 2009; Hafner et al. 2013; Pišlar et al. 2017, 2020, 2022; Allan et al. 2017; Horvat et al. 2025)
Cathepsin W	Endopeptidase-like cysteine cathepsin	Acidic lysosomal activity	Cytotoxic immune cells	Immune-cell-associated functions	Cystatins/stefins; compartmentalization	Limited direct CNS/glial evidence	(Kos et al. 2009; Turk et al. 2012; Pišlar and Kos 2014; Rawlings et al. 2018; Pišlar et al. 2021)

## The Roles of Cysteine Cathepsins in Microglia

Microglial cysteine cathepsins have emerged as important regulators of both physiological and pathological processes in the brain. Although primarily localized in lysosomes and involved in protein turnover, extensive research shows that these peptidases can also translocate to other cellular compartments or be secreted extracellularly, where they play additional roles in a wide range of microglial regulatory functions (reviewed in Hook 2006; Brix et al. 2008; Haque et al. 2008; Reiser et al. 2010; Turk et al. 2012; Pišlar and Kos 2014; Nakanishi 2020a; Pišlar et al. 2021; Stoka et al. 2023).

### Expression Patterns of Cysteine Cathepsins in Microglia

Alterations in the expression and distribution of cysteine cathepsins in microglia appear to be early pathological features of aging and neurodegenerative processes. Overall, the available evidence supports a consistent association between transitions in microglial functional states, formerly described as “activation”, and increased cathepsin expression; however, evidence varies among individual cathepsins. The most robust data are available for cathepsins B, S, and X, whereas other cathepsins, such as C, H, K, and L, remain less well characterized and are often studied only in experimental or disease contexts.

Certain cysteine cathepsins, e.g. B and C, are minimally expressed under physiological conditions but become strongly upregulated in activated microglia in aged mice, while others, notably S and X, are constitutively expressed and further show age-dependent increase in both expression and activity (Petanceska et al. 1996; Kos et al. 2005, 2009; Wendt et al. 2007, 2008; Obermajer et al. 2009; Koike et al. 2013; Pišlar et al. 2017, 2020; Wu et al. 2017). In parallel, changes in subcellular distribution indicate that ageing may also affect cathepsin trafficking and function rather than expression alone. For instance, the redistribution of cathepsin X from a predominantly lysosomal localisation to a more diffuse cytoplasmic pattern in aged mouse microglia points to potential alterations in proteolytic activity and substrate availability (Wendt et al. 2007, 2008). Together, these findings support the idea that increased cysteine cathepsin expression is part of a broader age-associated microglial state transition. However, it remains unclear whether this reflects adaptive responses to increased lysosomal demand or actively contributes to microglial dysfunction and chronic age-related neuroinflammation. Importantly, most evidence comes from animal models, which limits direct translation to human ageing.

A similar upregulation pattern is also associated with neurodegenerative pathologies. In AD, for example, senile plaque-associated microglia exhibit increased expression of cathepsins B (Mueller-Steiner et al. 2006), H, L (Ii et al. 1993; Fan et al. 2015; Gu et al. 2015) and X (Hafner et al. 2013), while cathepsins S and X are upregulated in microglia in degenerating brain regions in transgenic mouse models of amyotrophic lateral sclerosis (Wendt et al. 2007, 2008). These findings collectively suggest that cathepsin upregulation is also a common feature of disease-associated microglial reactivity. Nevertheless, the predominantly correlative nature of these data makes it difficult to distinguish whether increased cathepsin expression is a consequence of microglial state transition secondary to neuronal injury or an active contributor to disease progression.

Inflammatory stimulation further supports the link between microglial state transitions and increased cysteine cathepsin expression. For example, systemic injection of lipopolysaccharide (LPS) increased the expression and activity of cathepsins C (Fan et al. 2012) and X (Pišlar et al. 2020) in vivo. At the cellular level, LPS treatment altered the subcellular distribution of cathepsin X, as shown by decreased co-localization with the lysosomal marker LAMP1 and clathrin-containing vesicles in BV2 microglia, suggesting altered localization, trafficking, and secretion dynamics (Pišlar et al. 2017). This pattern extends across multiple inflammatory agents in addition to LPS, including interferon-γ, tumour necrosis factor (TNF)-α, interleukin (IL)−1β, and IL-6, which have been consistently shown to elevate the expression, activity, and extracellular secretion of cathepsins B, C, H, K, L, S, and X in vitro (Liuzzo et al. 1999; Gresser et al. 2001; Nakanishi 2003; Wendt et al. 2009; Fan et al. 2012, 2015; Pišlar et al. 2017, 2022; Allan et al. 2017; Xu et al. 2018b; Ni et al. 2019; Liu et al. 2019; Inoue et al. 2022; Zhang et al. 2025b). These data suggest that inflammatory treatment of microglia promotes not only increased cathepsin production but also changes in their intracellular localization and extracellular availability. However, much of the evidence relies on acute inflammatory stimulation or immortalized microglial cell lines, which oversimplifies the complex and heterogeneous states observed in vivo.

Overall, the available evidence supports a consistent association between aging, neuroinflammation, and neurodegeneration and increased cysteine cathepsin expression. Nevertheless, the strength of evidence varies among individual cathepsins and it remains unclear whether the observed changes accurately represent chronic neuroinflammatory conditions or instead reflect transient activation states, underscoring the need for studies in more physiologically relevant models.

### The Multifaceted Proteolytic Functions of Microglial Cysteine Cathepsins

Under physiological conditions, microglial cysteine cathepsins play an important role in maintaining protein homeostasis by degrading misfolded or damaged proteins. By contrast, most neurodegenerative diseases are characterized by endolysosomal dysfunction, often due to the accumulation of disease-specific, oligomeric, neurotoxic proteins, which drives microglial reactivity and upregulation of cysteine cathepsins (reviewed in Stoka et al. 2023). While this upregulation is often interpreted as adaptive, the available data suggest that it also reflects a functional shift in cathepsin activity rather than a simple increase in degradative capacity.

Several proteins implicated in neurodegenerative disorders have been identified as cathepsin substrates (reviewed in Stoka et al. 2023). A substantial number of studies support the involvement of cathepsins B, E, and S in amyloid precursor processing through intracellular β-secretase-like activity, contributing to downstream neurotoxicity in AD (Cataldo and Nixon 1990; Tagawa et al. 1991; Mackay et al. 1997; Hook et al. 2008, 2009, 2012; Schechter and Ziv 2011). At the same time, microglial cysteine cathepsins participate in Aβ clearance, a protective mechanism for resolving chronic inflammation and maintaining homeostasis in AD, highlighting a functional tension between production and degradation pathways (reviewed in Cappellano et al. 2013; Comi and Tondo 2017; Nakanishi 2020b). Notably, microglial cathepsin S, which retains its activity at a neutral pH outside lysosomes (Shi et al. 1994; Jordans et al. 2009), may also facilitate the extracellular clearance of intracellularly formed Aβ, which could influence Aβ levels during the early stages of aggregation and potentially modulate their neurotoxic effects (Pike et al. 1993). Particularly illustrative of this duality, cathepsin B exhibits context-dependent functions that reflect the balance between Aβ production and clearance (reviewed in Lowry and Klegeris 2018): in mouse models it has been shown to promote microglia-dependent Aβ accumulation in neurons (Wu et al. 2017), yet it is also essential for efficient microglial phagocytosis and degradation of Aβ, as in a recent study, its deficiency impaired clearance and exacerbated cognitive decline via disrupted PI3K/Akt signalling (Jiang et al. 2025), supporting disease-associated microglial functions. Furthermore, lysosomal damage during Aβ phagocytosis leads to the release of cathepsin B into the cytosol, activating NOD-leucine rich repeat and pyrin containing protein 3 (NLRP3) inflammasome and triggering IL-1β-mediated neuroinflammation in AD (Halle et al. 2008). These seemingly contradictory findings likely reflect differences in experimental context, with systemic inflammation-driven models capturing cathepsin B-dependent amplification of cytokine signalling and APP processing, whereas acute Aβ exposure models emphasize its role in microglial migration and phagocytic clearance. Thus, rather than representing opposing functions, cathepsin B appears to operate along two interconnected axes of Aβ metabolism depending less on its absolute expression levels than on its subcellular localization, lysosomal integrity, and the inflammatory state of microglia.

Beyond Aβ metabolism, cathepsins are also involved in CNS pathology through the cleavage of substrates with distinct neurobiological functions. For example, cathepsin X sequentially cleaves two C-terminal amino acids of γ-enolase, a glycolytic enzyme with neurotrophic-like activity, thereby abolishing its neuroprotective function (Obermajer et al. 2009). In a mouse model of AD, γ-enolase is upregulated in microglia near senile plaques; however, its neuroprotective effect against Aβ toxicity is reversed by cathepsin X (Hafner et al. 2013). This illustrates how increased cathepsin activity can reshape the functional landscape of the microenvironment by modifying key regulatory proteins.

Cathepsins also contribute to extracellular remodelling processes that influence microglial behaviour, but evidence from multiple studies indicates both functional overlap and context-specific specialization. Cathepsins B, L, and S are generally capable of degrading a wide range of ECM proteins (Maciewicz and Etherington 1988; Kirschke et al. 1989; Buck et al. 1992; Shi et al. 1992; Guinec et al. 1993; Petanceska et al. 1996; Kawada et al. 1997). However, cathepsin S is specifically distinguished by its stability and retained activity at neutral pH (reviewed in Kirschke and Wiederanders 1994; Turk et al. 2000), enabling effective degradation of ECM components in the CNS, such as neurocan and phosphacan (Maciewicz and Etherington 1988; Kirschke et al. 1989; Shi et al. 1992; Petanceska et al. 1996). In the context of microglia, however, invasion, migration, and the repertoire of matrix-degrading enzymes following CNS injury or disease are tightly regulated by the cells’ activation states and pathological context (Lively and Schlichter 2013). Cysteine cathepsins can support microglial migration to sites of injury (Lively and Schlichter 2013; Lowry and Klegeris 2018). For instance, cathepsin S deficiency impairs microglial activation and migration following facial nerve axotomy in mice, suggesting its protective role after neuronal injury (Hai et al. 2007). However, this extracellular proteolytic activity can also have detrimental effects. In experimental ischemia, increased activity and secretion of microglial cathepsin L degrades the microvascular matrix, compromising blood-brain barrier integrity and promoting oedema and microhaemorrhage (Gu et al. 2015). These seemingly divergent roles likely reflect differences in cellular targets, spatial localization, and pathological context rather than fundamentally opposing functions. Cathepsin S primarily acts in microglial remodelling and migration, supporting coordinated immune responses to localized injury, whereas cathepsin L, particularly when released extracellularly under acute ischemic stress, seems to target structural components of the neurovascular unit, leading to tissue damage.

**Fig. 2:**
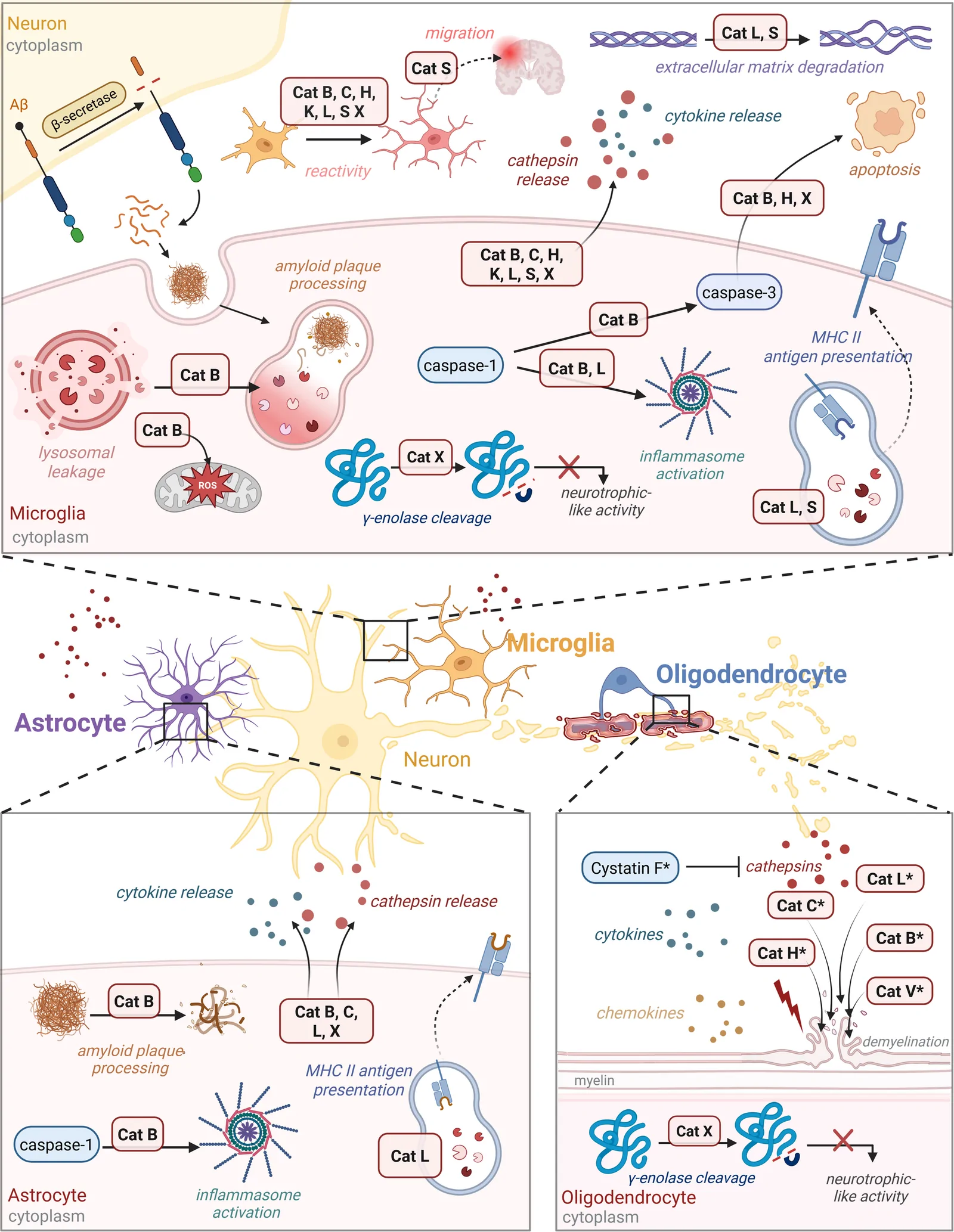
The roles of cysteine cathepsins in glial cells. Microglia-derived cysteine cathepsins participate in amyloid beta (Aβ) processing, antigen presentation, inflammasome activation, cytokine production, reactivity, extracellular matrix degradation, migration, and apoptosis. Astrocyte-derived cysteine cathepsins participate in Aβ processing, antigen presentation, inflammasome activation, and cytokine production. Oligodendrocyte-derived cathepsin X regulates oligodendrocyte differentiation. Additionally, cysteine cathepsins derived from cells of the CNS, such as microglia, astrocytes, and neurons (indicated by *), influence oligodendrocyte functions, including cytokine production, neuroinflammation, and myelination. Cat: cathepsin, MHC: major histocompatibility complex, ROS: reactive oxygen species. Created with BioRender

In summary, imbalances in the expression, activity, and secretion of microglial cathepsins can influence protein aggregation, cell migration, phagocytic efficiency, and neurotoxicity (Fig. 2; Table 2) (Frautschy et al. 1998). However, the available evidence does not support a single uniform role of microglial cathepsins. Instead, the overall impact of microglial cathepsins, either protective or detrimental, is highly context-dependent, and appears to depend on whether cathepsin activity supports lysosomal degradation, aggregate clearance, and phagocytosis, or whether their excessive secretion and mislocalization promote inflammatory signalling, extracellular matrix degradation, and neuronal injury. This distinction is important as cathepsin upregulation in disease models may reflect either a compensatory response to proteostatic stress or a direct contribution to neurotoxicity.

### Cysteine Cathepsins in Microglial Reactivity and Neuroinflammation

The molecular mechanisms driving microglial state transitions during neuroinflammation and neurodegeneration are complex and far from fully understood. Nonetheless, accumulating evidence highlights a pivotal role for cysteine cathepsins in these processes. Wendt et al. first demonstrated that cathepsins B, K, S, and X are upregulated intracellularly and secreted by BV2 microglial cells upon LPS stimulation, and that the conditioned medium from these activated cells is neurotoxic to the Neuro-2a neuroblastoma cell line (Wendt et al. 2009). Numerous in vitro studies have confirmed that microglial cathepsins B, H, K, L, S, and X are consistently upregulated and can be secreted into the extracellular compartment, promoting microglial responses with predominantly neurotoxic, pro-inflammatory functions, processes that have been traditionally described as neurotoxic (M1) polarization. Collectively, these studies support the idea that cathepsins act as amplifiers of pro-inflammatory signalling, at least in part through activating nuclear factor kappa-light-chain-enhancer of activated B cells (NF-κB), a transcription factor crucial for regulating immune and inflammatory responses, apoptosis, and cell stress (Wendt et al. 2009; Fan et al. 2015; Ni et al. 2015, 2019; Pišlar et al. 2017, 2022; Xu et al. 2018b; Liu et al. 2019; Zhao et al. 2021; Inoue et al. 2022; Zhang et al. 2025b).

For instance, in a mouse model of hypoxic-ischemic brain injury, elevated cathepsin B levels promoted neurotoxic microglial polarization through a cathepsin B-dependent autophagy mechanism involving NF-κB activation. Notably, this process operates within a broader proteolytic cascade, where early activation of the aspartyl peptidase cathepsin E precedes and promotes the late-stage induction of cathepsin B-dependent autophagy (Ni et al. 2015). In light of the current understanding of microglial biology, which rejects a strict binary classification, these findings are more appropriately interpreted as evidence that cysteine cathepsins support microglial functional states associated with enhanced neurotoxic activity. Importantly, deficiency or pharmacological inhibition of cysteine cathepsins has been shown to decrease microglial detrimental reactivity and promote neuroprotection, underscoring the potential of cysteine cathepsins as therapeutic targets in neuroinflammation-associated neurodegenerative diseases (Wendt et al. 2009; Fan et al. 2015; Ni et al. 2015, 2019; Pišlar et al. 2017, 2022; Xu et al. 2018b; Liu et al. 2019; Inoue et al. 2022; Zhang et al. 2025a, b). However, the reliability of this evidence varies considerably: while the role of cathepsin B is supported by multiple mechanistic and intervention studies, evidence for other cathepsins is often fragmentary or context-specific and derived from more limited or mode-specific observations. Moreover, much of this work relies on acute inflammatory paradigms, such as LPS stimulation, which may not fully recapitulate the complexity of chronic neuroinflammatory conditions.

Beyond their role in influencing microglial states, some studies also show that microglial cysteine cathepsins participate in antigen presentation. Cathepsins B, H, L, S, and X have been implicated in MHC II-dependent antigen presentation in antigen-presenting cells, either by processing antigens or regulating this process before or after their association with MHC II molecules (reviewed in Watts 1997; Riese and Chapman 2000). Among them, cathepsin S is particularly well supported as a regulator involved throughout the endocytic pathway, owing to its broad pH activity range (Riese and Chapman 2000). A study suggests its activity is essential for the degradation of proteins destined for the MHC II processing pathway and invariant chains in antigen-presenting cells, including microglia (Nakagawa et al. 1999). In contrast, the contribution of other cathepsins is less consistently defined and is often extrapolated from peripheral immune cells. Similarly, while cathepsins L and S have been implicated as key components in the regulation of the immune potential of microglia and are thought to be regulated by interferon-γ and TNF-α (Gresser et al. 2001), additional evidence is lacking. Cysteine cathepsins have also been linked to inflammasome activation and pro-inflammatory mediator production. Multiple studies have identified cathepsin B as a key mediator of caspase-1-dependent NLRP3 inflammasome activation, leading to IL-1β release (Hanamsagar et al. 2011; An et al. 2016; Xu et al. 2018a; Bai et al. 2018; Chevriaux et al. 2020). Additional cathepsins may contribute to inflammatory mediator production in a context-specific manner: for example, cathepsin C induces the expression of C-C motif chemokine ligand 2 and C-X-C motif chemokine ligand 2 (CXCL2) in microglia in a cryogenic brain lesion, thereby aggravating neuroinflammation (Zhao et al. 2021), and cathepsin B-mediated lysosomal dysfunction in aged microglial leads to mitochondrial damage, increased ROS production, and memory impairment (Ni et al. 2019). In addition, cathepsin L has been similarly shown to exacerbate neuroinflammation by promoting microglial M1 polarization and NLRP3 activation following spinal cord injury (Zhang et al. 2025a). However, while all pointing to inflammasome activation, many of these observations are derived from individual models, and comparative studies assessing the relative contributions of different cathepsins are lacking.

In connection with neurotoxic functions and neuroinflammation, microglial cysteine cathepsins have also been shown to initiate apoptotic signalling in microglia and contribute to microglia-mediated neuronal apoptosis. Cathepsin B is a central mediator in several contexts, including chromogranin A-induced microglial activation, where it promotes IL-1β maturation and secretion via caspase-1 (Terada et al. 2010) and induces neuronal apoptosis via caspase-3 activation (Kingham and Pocock 2001), thereby contributing to neuroinflammation and neurodegeneration. Similarly, in arsenic-induced microglial cell injury, upregulated cathepsin B activated the pro-apoptotic molecule Bid, triggering the lysosomal-mitochondrial apoptotic pathway (Zhang et al. 2023c). Other cathepsins, such as cathepsins H and X, have also been associated with neurotoxicity and microglial cell death: cathepsin H induced neuronal death in an in vivo model of LPS-induced neuroinflammation (Fan et al. 2015), and cathepsin X promoted microglial apoptosis and microglia-mediated neuronal damage (Pišlar et al. 2017). However, evidence for these is more limited and restricted to single experimental paradigms. This raises the possibility that some cathepsin-dependent effects reflect context-specific stress responses rather than general mechanisms of neuroinflammation and neurodegeneration.

Nevertheless, microglial studies provide the strongest evidence for the involvement of glial cysteine cathepsins in microglial state transitions and neuroinflammation, suggesting their multifaceted and highly context-dependent roles in microglial biology (Fig. 2; Table 2). However, the strength of evidence differs between individual cathepsins and experimental models. While cathepsin B is supported by multiple in vitro studies, evidence for other discussed cathepsins often relies solely on immortalized cell lines, disease-specific models, or use of pharmacological inhibitors, with limited integration across systems. Notably, only a recent study by Jiang et al. has examined cysteine cathepsins in accordance with the novel nomenclature guidelines and linked their expression to DAM (Jiang et al. 2025). Future work should therefore clarify how individual cathepsins function across specific microglial states (e.g. disease-associated, phagocytic, inflammatory, and repair-associated microglia), whether they merely accompany DAM-like states or actively regulate transitions between the different proposed microglial states. Also, they should determine how their roles differ between acute injury, chronic neuroinflammation, aging, and specific neurodegenerative diseases. A key unresolved question is whether increased cathepsin activity drives harmful microglial responses, reflects an adaptive lysosomal response to disease-associated stress, or both, depending on disease stage and localization. Further open questions concern functional redundancy versus specialization among cathepsins, the relative contributions of intracellular versus extracellular activity, and the degree to which findings from acute models translate to chronic neurodegeneration.

**Table 2:** Overview of the functions of cysteine cathepsins in glial cells

Glial cell type	Cysteine cathepsin	Function	References
**Microglia**	Cathepsin B	Indirectly induces microglia-dependent Aβ accumulation in neurons via β-secretase activity	(Wu et al. 2017)
		Promotes Aβ phagocytic clearance in Alzheimer’s disease	(Cappellano et al. 2013; Comi and Tondo 2017; Nakanishi 2020b; Jiang et al. 2025)
		Activates the NLRP3 inflammasome and triggers IL-1β release via caspase-1	(Halle et al. 2008; Terada et al. 2010; Hanamsagar et al. 2011; An et al. 2016; Xu et al. 2018a; Bai et al. 2018; Chevriaux et al. 2020)
		Promotes neurotoxic polarization and neuroinflammation via NF-κB activation	(Wendt et al. 2009; Ni et al. 2015, 2019; Inoue et al. 2022)
		Impairs memory by degrading mitochondrial transcription factor A and producing reactive oxygen species and pro-inflammatory mediators	(Ni et al. 2019)
		Induces neuronal apoptosis via caspase-3 activation	(Kingham and Pocock 2001)
		Triggers apoptosis through Bid activation	(Zhang et al. 2023c)
	Cathepsin C	Promotes neurotoxic polarization and neuroinflammation via protein kinase C/p38MAPK/NF-κB activation	(Liu et al. 2019; Zhao et al. 2021)
		Enhances neuroinflammation by inducing C-C motif chemokine ligand 2 and C-X-C motif chemokine ligand 2	(Zhao et al. 2021)
	Cathepsin H	Promotes neurotoxic polarization, neuroinflammation, and neuronal death	(Fan et al. 2015)
	Cathepsin K	Promotes neurotoxic polarization and neuroinflammation	(Wendt et al. 2009)
	Cathepsin L	Disrupts microvessel integrity via extracellular matrix degradation	(Gu et al. 2015)
		Promotes neurotoxic polarization and neuroinflammation via NF-κB activation	(Xu et al. 2018b; Inoue et al. 2022; Zhang et al. 2025a)
		Activates the NLRP3 inflammasome	(Zhang et al. 2025a)
		Mediates major histocompatibility complex II antigen presentation	(Gresser et al. 2001)
	Cathepsin S	Promotes microglial activation and migration via extracellular matrix degradation	(Hai et al. 2007; Lively and Schlichter 2013; Lowry and Klegeris 2018)
		Promotes neurotoxic polarization and neuroinflammation via NF-κB activation	(Wendt et al. 2009; Zhang et al. 2025b)
		Processes the invariant chain to enable major histocompatibility complex II antigen presentation	(Nakagawa et al. 1999; Riese and Chapman 2000; Gresser et al. 2001)
	Cathepsin X	Abolishes γ-enolase neuroprotection against Aβ and lipopolysaccharide via proteolytic cleavage	(Obermajer et al. 2009; Hafner et al. 2013; Pišlar et al. 2017)
		Promotes neurotoxic polarization, apoptosis, and neuroinflammation via PI3K/Akt activation	(Wendt et al. 2009; Pišlar et al. 2017, 2020, 2022)
**Astrocytes**	Cathepsin B	Degrades Aβ and generates Aβ peptides	(Oberstein et al. 2020)
		Activates the NLRP3 inflammasome and triggers IL-1β release via caspase-1	(Qiao et al. 2016)
	Cathepsin C	Enhances neuroinflammation by inducing C-X-C motif chemokine ligand 2	(Zhao et al. 2021)
	Cathepsin K	Alters astrocyte number and phenotype	(Dauth et al. 2011)
	Cathepsin L	Mediates major histocompatibility complex II antigen presentation	(Gresser et al. 2001)
	Cathepsin S	Contributes to sensory impairment during neuroinflammation in EAE	(Kim et al. 2020)
**Oligodendrocytes**	Cathepsin X	Impairs oligodendrocyte maturation and differentiation	(Horvat et al. 2025)
*Cysteine cathepsins derived from microglia, astrocytes, and neurons affecting oligodendrocyte function.	Cathepsin B*	Impairs remyelination and promotes oligodendrocyte and neuronal damage	(Ma et al. 2007)
	Cathepsin C*	Enhances demyelination in EAE	(Durose et al. 2019)
		Enhances demyelination by inducing C-X-C motif chemokine ligand 2	(Liang et al. 2016)
		Produces toxic tumour necrosis factor-α and protective IL-1β and impairs myelin integrity Enhanhes T-cell expansion and tertiary lymphoid structures, resulting in earlier onset and more severe chronic EAE	(Shimizu et al. 2017) (Liu et al. 2025)
	Cathepsin H*	Contributes to EAE development	(Okada et al. 2018)
		Impairs remyelination and promotes oligodendrocyte and neuronal damage	(Ma et al. 2007)
	Cathepsin L*	Contributes to EAE development	(Allan and Yates 2015)
		Impairs remyelination and promotes oligodendrocyte and neuronal damage	(Ma et al. 2007)
	Cathepsin X*	Promotes IL-1β and neuroinflammation in EAE	(Allan et al. 2017)
	Cathepsin V*	Enhances demyelination	(Shimizu et al. 2017)

Aβ: amyloid beta, IL: interleukin, EAE: experimental autoimmune encephalomyelitis, NF-κB: nuclear factor kappa-light-chain-enhancer of activated B cells, NLRP3: NOD-leucine rich repeat and pyrin containing protein 3

## The Roles of Cysteine Cathepsins in Astrocytes

As multifunctional glial cells, astrocytes are essential for maintaining CNS homeostasis and responding to injury. Increasing evidence suggests that cysteine cathepsins contribute to astrocyte-mediated neuroinflammatory and neurodegenerative processes. Under pathological conditions, the expression and activity of these peptidases are dynamically regulated, indicating that astrocyte-derived cysteine cathepsins may participate in Aβ handling, antigen presentation, inflammasome activation, and chemokine production. However, compared with microglia, their causal contribution to neuroinflammation and neurodegeneration remains less well defined, and available evidence often comes from specific disease models or mixed-cell experimental systems (Pišlar and Kos 2014; Liddelow et al. 2020; Pišlar et al. 2021; Patani et al. 2023).

### Expression Patterns of Cysteine Cathepsins in Astrocytes

Distinct expression patterns and functional roles of cysteine cathepsins have been observed in astrocytes across various CNS pathologies, however, the available evidence remains fragmented and largely based on descriptive studies in heterogeneous models. In neurodegenerative conditions, astrocyte cathepsin upregulation is most consistent. Multiple reports consistently show increased cathepsin B expression in reactive astrocytes in anterior horns of amyotrophic lateral sclerosis patients (Kikuchi et al. 2003), as well as in MS (Bever and Garver 1995) and AD (Nakamura et al. 1991; Ii et al. 1993) patients. Similar upregulation has been observed in rats under neuropathological conditions induced by the neurodegenerative agent kainic acid or neurotoxic cobalt pellet implants in the brain (Bernstein et al. 1986), suggesting that astrocyte cathepsin B induction is a common response to neurodegenerative and neurotoxic stress. Additionally, elevated levels of cathepsins H, L, and S have been detected in astrocytes associated with senile plaques in AD brain tissue (Ii et al. 1993; Lemere et al. 1995). Conversely, findings from demyelinating and neuroinflammatory models are less consistent. In a murine model of demyelination, cathepsins B, H, and L were only weakly expressed in astrocytes, whereas the cathepsin inhibitor cystatin F was broadly expressed (Ma et al. 2007). Similarly, in the olfactory bulb of experimental autoimmune encephalomyelitis (EAE) mice at the paralytic stage, cathepsins B, C, H, S, W, and X were all detected, with cathepsins C and S showing the most pronounced upregulation (Kim et al. 2020). These observations highlight that astrocyte cathepsin expression is not uniform but varies substantially on disease model, stage and inflammatory environment. Single studies also report observation of cathepsin K in astrocytes both in mice (Dauth et al. 2012) and schizophrenic patients (Lendeckel et al. 2009). Moreover, reactive astrocytes express cathepsin X in the brain in an LPS-induced neuroinflammation model (Pišlar et al. 2020) and the cortex of aged mice (Wendt et al. 2007). Finally, cathepsin L has been confirmed in primary cultures of mouse astrocytes (Gresser et al. 2001), and a proteomic analysis revealed that primary astrocytes secrete cathepsins B, L, and X, implying that, similar to those of microglia, astrocyte-derived cysteine cathepsins may exert important non-lysosomal functions in the CNS (Greco et al. 2010). Overall, these findings indicate that astrocyte cathepsin expression is a common feature of reactive states but varies markedly across disease contexts, with the strongest and most consistent induction in neurodegeneration.

### The Roles of Cysteine Cathepsins in Modulating Astrocyte States

Astrocyte reactivity is a dynamic process characterized by morphological, molecular, and functional changes in response to various stimuli (reviewed in Zuchero and Barres 2015; Liddelow et al. 2017; Fan and Huo 2021; Singh 2022; Patani et al. 2023). While cathepsin D, an aspartyl peptidase, has been implicated in the reactivity of both microglia and astrocytes in the murine CNS (Suzuki et al. 2022), the involvement of cysteine cathepsins in astrocyte reactivity remains largely unexplored. Existing evidence is limited and derives primarily from genetic models. Dauth et al. reported that cathepsin K-deficient mice exhibited altered expression of glial fibrillary acidic protein (a marker of reactive astrocytes), astrocyte number, and process morphology. These changes were accompanied by alterations in oligodendrocytes and microglia, making it difficult to disentangle astrocyte-specific effects from broader glial interactions (Dauth et al. 2011). Furthermore, astrocyte-rich cultures derived from cathepsin L-deficient mouse brains exhibited enhanced oligodendrocyte maturation without major changes in astrocyte metabolic support to other cell types, suggesting indirect or context-dependent roles (Dauth et al. 2012). Mechanistically, cysteine cathepsin dysfunction may lead to the accumulation of misfolded proteins, potentially triggering astrocyte reactivity and promoting chronic inflammation and neuronal impairment (reviewed in Pišlar et al. 2021; Liu et al. 2024). Given the established role of cysteine cathepsins in microglial reactivity, it is plausible that these peptidases may also contribute to the molecular mechanisms driving astrocyte state transitions. Nonetheless, current evidence is sparse and lacks direct functional validation. Future studies should clarify whether these enzymes influence astrocyte states and, if so, whether they directly drive state switching or instead modulate it indirectly through effects on cellular stress, lysosomal function, or interactions with other glial cell types.

### Astrocyte-Derived Cysteine Cathepsins in Neuroimmune Regulation

Astrocytes play an active role in the brain’s immune response, and cysteine cathepsins have emerged as modulators of these functions. A study by Oberstein et al. suggests that although astrocytes are not professional phagocytes, they exhibit weak phagocytic activity and participate in Aβ handling in cellular models of AD. In this context, the authors report that cathepsin B exerts opposing effects depending on its localisation: lysosomal cathepsin B is involved in the degradation of Aβ, whereas membrane-associated cathepsin B produces N-terminally truncated Aβ peptides (Oberstein et al. 2020), illustrating how subcellular distribution can shape functional outcomes. Beyond protein turnover, astrocyte-derived cathepsin B has been implicated in actively promoting neuroinflammation. Conditions that induce lysosomal membrane permeabilization, such as mutations in Atp13a2 (a gene encoding a transmembrane lysosomal ATPase) or exposure to 1-methyl-4-phenylpyridinium (MPP+), promote the release of cathepsin B into the cytosol, leading to activation of the NLRP3 inflammasome and caspase-1, promoting IL-1β production (Qiao et al. 2016). Additional studies implicate other cathepsins in neuroinflammatory responses. For example, cathepsin S is upregulated in astrocytes in a mouse model of EAE and associated with transient olfactory dysfunction (Kim et al. 2020), while cathepsin C promotes CXCL2 production in both microglia and astrocytes in a cryogenic brain lesion model (Zhao et al. 2021). Together, these findings suggest that astrocyte cathepsins may amplify inflammatory signalling, although, once again, most evidence derives from specific models. Another study implies that cysteine cathepsins also contribute to antigen processing in astrocytes: cathepsins L and S are supposed to regulate invariant chain degradation during MHC II trafficking and maturation, as decreased cathepsin L activity and the absence of cathepsin S impaired antigen presentation capacity. Moreover, astrocytes display lower antigen-presenting capacity compared to microglia, a difference attributed to the cell-specific regulation of the expression and activity of cathepsins L and S under neuroinflammatory conditions (Gresser et al. 2001).

To summarize, the available evidence suggests important roles for astrocyte-derived cysteine cathepsins as key modulators ofastrocyte function, with roles extending beyond lysosomal proteolysis, including antigen presentation, inflammasome activation, and potentially state modulation (Fig. 2; Table 2). However, compared to microglia, their functions remain less well characterized, and the current evidence is still very limited and often model-specific, with the lack of several studies pointing to the same function. At present, it remains difficult to determine whether cysteine cathepsins actively drive astrocyte functions or mainly reflect lysosomal remodelling in reactive states. Addressing this will require astrocyte-specific genetic models, validation in primary human astrocytes, and approaches such as single-cell and spatial analyses to resolve cell-autonomous functions and determine whether astrocyte-derived cathepsins represent viable therapeutic targets.

## The Roles of Cysteine Cathepsins in Oligodendrocytes

In contrast to microglia and astrocytes, the current understanding of the role of oligodendrocyte-derived cysteine cathepsins is limited, especially regarding their intracellular functions. Conversely, growing evidence suggests that cysteine cathepsins secreted by microglia and astrocytes significantly influence oligodendrocyte health and function, affecting processes such as myelin integrity, remyelination, and cell survival (Deng et al. 2008; Fischer et al. 2014; Moore et al. 2015; Boccazzi et al. 2022). Therefore, in addition to examining currently proposed intracellular roles of cysteine cathepsins in oligodendrocytes, attention is also given to how cathepsins released from neighbouring cells modulate oligodendrocyte function, particularly in the context of myelination.

### Expression Patterns of Cysteine Cathepsins in Oligodendrocytes

In oligodendrocytes, cysteine cathepsins are less commonly described that in astrocytes and especially microglia. Cathepsin K has been detected in white matter oligodendrocytes in the brains of schizophrenic patients (Lendeckel et al. 2009). In the aged mouse brain, cathepsin X is abundant in microglia and astrocytes but barely detectable in oligodendrocytes, indicating distinct expression patterns between glial cell types (Wendt et al. 2007). Consistent with this, the expression of this peptidase has been confirmed in the human oligodendroglioma cell line and is lower in differentiated oligodendrocytes (Horvat et al. 2025), suggesting regulation during maturation. Taken together, these observations possibly suggest a more restricted and tightly regulated cathepsin profile in oligodendrocytes compared to other glial cells, which is particularly relevant when considering the involvement of cysteine cathepsins in myelination, but maybe also point to the relative lack of research in this cell type.

### The Roles of Cysteine Cathepsins in Demyelination and Remyelination

Demyelination, accompanied by oligodendrocyte loss, axonal damage, and degeneration, is a hallmark of several neuroinflammation-associated neurodegenerative diseases, such as MS (reviewed in Lassmann et al. 2012; Höftberger and Lassmann 2017; Zveik et al. 2024). Peptidases, including cysteine cathepsins, have been implicated in the pathogenesis of these disorders (reviewed in Hirsch 1981; Bever and Whitaker 1985; Klotz et al. 2023; Depp et al. 2023); however, the expression and function of cysteine cathepsins in oligodendrocytes remain poorly defined. Instead, most current studies focus on microglia and astrocytes as sources of elevated peptidase levels during demyelination (Hirsch 1981), and show that cysteine cathepsins released from these glial cells influence oligodendrocyte function and myelination (Fig. 2; Table 2).

Insights into the complex and context-dependent functions of individual cathepsins have largely been derived from myelin oligodendrocyte glycoprotein (MOG)-induced EAE, a widely used model of demyelination. Studies using this model demonstrate that cathepsins B, L, and S exhibit functional redundancy in antigen processing, as deficiency in individual enzymes in a mouse model of EAE did not impair MOG presentation to CD4 + T-cells. However, cathepsin L-deficient mice did not develop EAE, suggesting non-redundant functions in disease progression (Allan and Yates 2015). Additional cathepsins have been shown to contribute to disease severity. Cathepsin C, for instance, exacerbates demyelination when overexpressed in microglia in MOG-induced EAE, whereas its absence mitigates disease severity (Durose et al. 2019). This is supported by recent findings showing that cathepsin C promotes T-cell expansion and the formation of tertiary lymphoid structures in the CNS, leading to earlier onset and more severe chronic MOG-induced EAE (Liu et al. 2025). Furthermore, cathepsin H has been implicated in the early onset of EAE (Okada et al. 2018), and cathepsin X deficiency reduced neuroinflammation and IL-1β levels in EAE without affecting MOG processing or CD4 + T-cell activation (Allan et al. 2017). High-throughput sequencing and omics-based studies further extend these observations by identifying upregulation of cathepsin F in paediatric patients with MOG-associated disease (a CNS autoimmune demyelinating disorder) (Wang et al. 2024) and dysregulated microglial cathepsin L in patients with MS (Drake et al. 2023). While these datasets strengthen the association between cathepsins and demyelinating pathology, they are largely correlative and do not resolve whether these changes are causative, compensatory, or cell-type specific. Together, cysteine cathepsins from non-oligodendrocyte CNS cell types appear to act mainly through modulation of immune and inflammatory pathways rather than direct regulation of oligodendrocyte function. Importantly, these effects may not be uniformly detrimental. For instance, cathepsin C promotes the production of TNF-α, which is highly toxic to oligodendrocytes, but also IL-1β, which can support remyelination (reviewed in Shimizu et al. 2017). Thus, although cysteine cathepsins are predominantly linked to exacerbation of demyelination, their functions remain complex and shaped by the inflammatory context and timing.

After the onset of myelin degeneration, efficient clearance of myelin debris is essential for effective remyelination. This process is also partly mediated by cysteine cathepsins, while their endogenous inhibitors, such as cystatin F (Ma et al. 2011), protect the CNS once debris clearance is complete. Thus, a balance between peptidases and their inhibitors is crucial for controlling both demyelination and remyelination. Cystatin F is typically absent in the healthy brain but becomes highly expressed in activated microglia and reactive astrocytes during acute demyelination (Ma et al. 2011). In a demyelinating mouse model, microglia upregulated cathepsins B, H, and L, whereas astrocytes produced cystatin F, suggesting a spatial division of proteolytic activity and its regulation. As demyelination progressed, the increasing microglia-to-astrocyte ratio indicated a shift of this balance toward elevated proteolytic activity, implicating that inadequate control of cathepsin activity may hinder remyelination and contribute to oligodendrocyte and neuronal damage (Ma et al. 2007). This concept is further supported by observations in both mouse models of impaired myelination and spinal cord tissues from MS patients, where cystatin F expression overlapped with regions of active remyelination, but was reduced in chronic demyelination, coinciding with impaired remyelination (Ma et al. 2011). Cathepsin C, another target of cystatin F, further illustrates the complexity of this regulatory axis: in a cuprizone-induced demyelination mouse model, its activity exacerbates demyelination in the presence of cystatin F, likely due to the increased expression of the glia-derived chemokine CXCL2 and subsequent inflammatory cell recruitment, an effect reversed by cathepsin C knockdown (Liang et al. 2016). Functional studies reinforce the importance of this regulatory system. Enhanced demyelination has been observed following cystatin F knockdown or cathepsin V overexpression, whereas inhibition of cathepsin C preserved myelin integrity in Plp^4e/−^ mice with impaired remyelination (Shimizu et al. 2017). Notably, evidence for direct, cell-autonomous roles for cysteine cathepsins in oligodendrocytes remains limited. Cathepsin X is currently the only cysteine cathepsin identified to date with a defined intracellular role in these cells, where it impairs their maturation and differentiation by abolishing the neurotrophic activity of γ-enolase (Obermajer et al. 2009; Horvat et al. 2025). This contrasts with the substantial body of work describing cathepsin activity in microglia and astrocytes, emphasizing an imbalance in the current understanding of their roles across cell types.

In summary, accumulating evidence indicates that oligodendrocyte function is significantly influenced by cathepsins secreted from microglia and astrocytes, which modulate myelin integrity, demyelination, remyelination, and cell survival (Fig. 2; Table 2). Nevertheless, interpretation of these findings is constrained by the predominant use of complex in vivo disease models, in which multiple cell types are simultaneously activated and interact dynamically, making it difficult to distinguish direct effects on oligodendrocytes from secondary immune or glial responses. In contrast, intrinsic roles of oligodendrocyte-derived cysteine cathepsins are only beginning to emerge. Addressing this gap will require future studies to clarify their intracellular functions in oligodendrocyte precursor cells and mature oligodendrocytes, to determine how their expression and activity change during differentiation, demyelination and remyelination, and to establish whether and how their dysregulation contributes to demyelinating diseases. Such insights will be essential for evaluating whether cysteine cathepsins could be targeted therapeutically to promote remyelination and preserve oligodendrocyte health without compromising myelin debris clearance.

## Glial Cysteine Cathepsins as Therapeutic Targets in Neuroinflammation

Neurodegenerative diseases pose a major global health challenge, as their incidence and prevalence are increasing with population aging. Despite considerable advances in understanding their molecular and cellular underpinnings, effective disease-modifying therapies remain limited, underscoring the urgent need for novel strategies (reviewed in Agnello and Ciaccio 2022; Bhat and Dhaneshwar 2024). Neuroinflammation is a common pathological feature across diverse neurodegenerative diseases, and its modulation offers a promising avenue to mitigate the onset and progression of neurodegeneration (reviewed in Dorothée 2018; Zhang et al. 2023b; Shi and Yong 2025). Various approaches have been proposed, including the inhibition of pro-inflammatory cytokines, chemokines and their receptors, inflammasome activity, and peptidases; modulation of microglial and astrocyte reactivity; and promotion of anti-inflammatory signalling (reviewed in Fan and Huo 2021; Guo et al. 2022; Zhang et al. 2023b).

Given the central roles of peptidases, including cysteine cathepsins, in glial cell function and neuroinflammation, and their excessive proteolytic activity and extracellular secretion under pathological conditions, glial cysteine cathepsins have emerged as potential, but still preclinical therapeutic targets in neuroinflammation and neurodegeneration (reviewed in Pišlar et al. 2021). Under physiological conditions, cysteine cathepsin enzymatic activity is tightly regulated by endogenous inhibitors such as cystatins. However, in CNS diseases, these inhibitors are often downregulated and thus cannot restrain excessive cathepsin activity. Moreover, as cystatins function as broad-spectrum inhibitors, their therapeutic use would likely result in substantial off-target effects (reviewed in Pišlar and Kos 2014). Consequently, research has focused on the development of reversible, selective, small-molecule inhibitors that can permeate the blood-brain barrier and suppress the excessive proteolytic activity of cysteine cathepsins associated with neuroinflammation and neurodegeneration (reviewed in Pišlar and Kos 2014).

Several in vitro and in vivo studies have examined the effects of cysteine cathepsin inhibition on neuroinflammation and neurodegeneration. A pivotal study by Wendt et al. demonstrated that intracellular inhibition of cysteine cathepsins in cultured BV2 microglia with the broad-spectrum inhibitor E64-d or the cathepsin B-selective inhibitor CA-074Me decreases pro-inflammatory cytokine expression and attenuates neurotoxicity. Conversely, extracellular inhibition exerted no protective effects (Wendt et al. 2009). Their findings indicate that only intracellular inhibition of cysteine cathepsins decreases neurotoxic microglial activation. Subsequent studies confirmed that pharmacological inhibition of intracellular cathepsins B, K, L, S, and X with membrane-permeant inhibitors decreases neurotoxic microglial activation, limits microglial apoptosis, and promotes neuroprotection (Wendt et al. 2009; Fan et al. 2015; Ni et al. 2015, 2019; Pišlar et al. 2017, 2022; Xu et al. 2018b; Liu et al. 2019; Inoue et al. 2022; Zhang et al. 2025a, b). However, most of these findings remain preclinical and are derived from cell culture systems or animal models, so their predictive value for human neurodegenerative diseases remains to be established.

Cathepsins B and L have been implicated as mediators of astrocyte injury in a rat model of ischemic stroke. Their inhibition in astrocytes with the selective cathepsin B inhibitor CA-074Me and selective cathepsin L inhibitor CLICK148 conferred neuroprotection by blocking tBid-mediated mitochondrial apoptotic signalling (Xu et al. 2014). Moreover, specific inhibition of cathepsin B in astrocytes attenuated the activation of NLRP3 inflammasomes and caspase-1, decreasing neuroinflammation in a Parkinson’s disease model (Qiao et al. 2016). In a rat model of neuroinflammation-induced neurodegeneration, cathepsin X was strongly upregulated in activated microglia and reactive astrocytes, and its inhibition with AMS36 produced neuroprotective effects (Pišlar et al. 2020). These studies support the therapeutic relevance of astrocyte- and microglia-associated cathepsins, but they also emphasize that inhibitor effects are model-, cell type-, and disease-stage-dependent.

Allan and Yates investigated the effects of the inhibitor LHVS on oligodendrocytes. Although LHVS has been described as a selective cathepsin S inhibitor, their study demonstrated that it also successfully decreases the activity of cathepsins B and L. The observation that both wild-type and cathepsin S-deficient mice exhibited decreased MHC II surface expression, MOG antigen presentation, and EAE severity suggests that LHVS-mediated inhibition of multiple cysteine cathepsins may be required for protection against EAE (Allan and Yates 2015). Furthermore, inhibition of cathepsin X with AMS36 altered cell morphology and promoted oligodendrocyte differentiation by preserving the active form of γ-enolase (Obermajer et al. 2009; Horvat et al. 2025). Finally, in animal AD models, inhibition of the β-secretase activity of cathepsin B with CA-0174Me and E-64d delayed memory loss and decreased Aβ plaque burden (Hook et al. 2008, 2011, 2014). These effects may at least partly reflect inhibited cathepsin B activity in glial cells (Inoue et al. 2022). Further studies are needed to determine whether comparable target engagement, efficacy, and safety can be achieved in the human CNS.

As previously noted, cysteine cathepsins fulfil essential physiological roles. Consequently, despite evidence of beneficial effects in certain contexts, their inhibition is not universally beneficial. Moreover, under particular conditions, not only preserving but even enhancing cysteine cathepsin activity may provide therapeutic benefits. Hwang et al. reported that early cathepsin B upregulation can decelerate the development of dementia by enhancing protein clearance in neurons, thereby improving synaptic function and cognition (Hwang et al. 2019). Analogously, enzyme replacement strategies have been investigated to boost lysosomal activity and facilitate the clearance of aggregation-prone substrates. For example, intracranial administration of recombinant cathepsins B and L promotes substrate clearance and decreases neuroinflammation (reviewed in Drobny et al. 2022). However, the evidence remains limited, and because these interventions were not glial cell-specific, the observed protective effects cannot be conclusively attributed to glial cathepsin activity.

Given these findings, therapeutic strategies targeting cysteine cathepsins in neuroinflammation and neurodegeneration face several important challenges. To date, although cathepsin inhibitors have shown promising effects in vitro and in animal models, none has yet advanced to clinical trials in these indications (Di Matteo et al. 2025). Notably, in other disease areas such as cancer and inflammatory disorders, they have reached clinical testing and demonstrated therapeutic potential, but their translation has often been limited by adverse effects and insufficient specificity. Several factors contribute to these limitations. First, the structural similarity of catalytic sites among cathepsins complicates the development of selective inhibitors and increases the risk of cross-reactivity and off-target effects (Siklos et al. 2015; Senjor et al. 2023; Di Matteo et al. 2025). Second, due to their essential physiological roles, broad or sustained inhibition may interfere with protective cellular pathways (Turk et al. 2012; Yadati et al. 2020; Pišlar et al. 2021). While targeted delivery approaches, such as antibody-drug conjugates, may enhance specificity and reduce systemic toxicity, their application in CNS diseases is limited by another major challenge: the blood-brain barrier restricts the entry of many small molecules and biologics into the brain parenchyma. Although this barrier can be bypassed by intrathecal administration, such an approach is highly invasive and unsuitable for long-term therapy. Therefore, alternative strategies that enable CNS access following systemic administration, such as receptor-mediated transcytosis (an example of “brain shuttle” systems), may represent a promising approach (Abbott et al. 2010; Wu et al. 2023; Prades et al. 2025; Di Matteo et al. 2025). Overall, successful clinical translation will require the development of reversible inhibitors that selectively target the pathological activities of individual cathepsins while preserving their physiological functions. Therapeutic strategies must be carefully tailored to the pathological context, with success depending on precise modulation of cathepsin activity in a cell type- and disease stage-specific manner (reviewed in Pišlar and Kos 2014; Senjor et al. 2023; Prades et al. 2025).

## Conclusions

Microglia, astrocytes, and oligodendrocytes collectively shape both physiological and pathological processes in the CNS, including neuroinflammation and neurodegeneration. In these cells, cysteine cathepsins seem to contribute to a wide range of cellular mechanisms beyond lysosomal proteolysis. Dysregulation of these peptidases is closely associated with pathological processes underlying neurodegenerative diseases. Understanding the distinct roles of individual cysteine cathepsins in these processes is therefore critical for elucidating the molecular bases of neuroinflammation-associated neurodegeneration. Current evidence highlights the need for further investigation, particularly into the functions of cysteine cathepsins in astrocytes and oligodendrocytes. Importantly, future studies should also consider emerging insights into the complex and dynamic states of microglia and astrocytes. Although modulating glial cysteine cathepsins holds therapeutic potential to mitigate neurodegeneration, achieving selective targeting without disrupting their essential physiological roles remains a major challenge. Successful translation will require intense further research, including the development of specific delivery strategies that must include precise timing and cellular context to ensure safe and effective interventions for neuroinflammatory and neurodegenerative diseases.

## Data Availability

No datasets were generated or analysed during the current study.
